# Splenic volume as a predictor of survival in cancer patients treated with immune checkpoint inhibitors

**DOI:** 10.3389/fimmu.2025.1598484

**Published:** 2025-05-30

**Authors:** Yunhua Zhang, Xin Fu, Lilong Zhang, Qing Zhou, Weixing Wang

**Affiliations:** ^1^ Department of Ultrasound Imaging, Renmin Hospital of Wuhan University, Wuhan, Hubei, China; ^2^ Hubei Key Laboratory of Digestive System Disease, Renmin Hospital of Wuhan University, Wuhan, Hubei, China; ^3^ General Surgery Laboratory, Renmin Hospital of Wuhan University, Wuhan, Hubei, China; ^4^ Department of Anesthesiology, Renmin Hospital of Wuhan University, Wuhan, Hubei, China; ^5^ Department of General Surgery, Renmin Hospital of Wuhan University, Wuhan, Hubei, China

**Keywords:** immune checkpoint inhibitors, cancer, splenic volume, prognosis, hepatocellular carcinoma

## Abstract

**Objective:**

This investigation seeks to examine the association between spleen volume and prognosis in cancer patients undergoing immune checkpoint inhibitor (ICI) treatment.

**Methods:**

We performed a retrospective analysis involving 61 patients diagnosed with hepatocellular carcinoma (HCC) who received ICIs at our institution. We evaluated the relationship between baseline splenic volume and its changes during ICI therapy concerning overall survival (OS) and progression-free survival (PFS) using a log-rank test. To identify relevant literature, we searched databases such as PubMed, EMBASE, the Cochrane Library, and Google Scholar up until February 20, 2024. The primary metrics assessed were hazard ratios (HR) for both OS and PFS, with pooled estimates and corresponding 95% confidence intervals (CIs) calculated.

**Results:**

Within our study population, findings demonstrated a significantly decreased OS (HR: 2.02, 95% CI: 1.08–3.77, *p* = 0.027) and PFS (HR: 1.84, 95% CI: 1.05–3.21, *p* = 0.032) in HCC patients with a high baseline spleen volume, compared to individuals with lower spleen volumes. Additionally, HCC patients who experienced an increase in spleen volume during ICI therapy exhibited significantly poorer OS (HR: 2.27, 95% CI: 1.17–4.41, *p* = 0.016) and PFS (HR: 2.40, 95% CI: 1.30–4.41, *p* = 0.005) than those whose spleen volume decreased. The meta-analysis results revealed that subjects with higher spleen volumes had a significantly reduced OS (HR: 1.74, 95% CI: 1.12–2.72, *p* = 0.014) and PFS (HR: 1.35, 95% CI: 1.15–1.58, *p* < 0.001) compared to counterparts with lower volumes. Furthermore, the data clearly highlighted that patients with increases in splenic volume faced significantly poorer clinical outcomes, as indicated by reduced OS (HR: 1.83, 95% CI: 1.36–2.46, *p* < 0.001) and PFS (HR: 1.70, 95% CI: 1.28–2.25, *p* < 0.001) relative to those with decreases in splenic size.

**Conclusion:**

A higher baseline spleen volume and an increase in spleen volume during ICI therapy were predictors of a poor prognosis in cancer patients treated with ICI.

## 1 Introduction

Immune checkpoints, which encompass both stimulatory and inhibitory signals, regulate the immune response and protect neoplastic cells from immune detection (1–3). In recent years, oncology has experienced notable progress, highlighted by the emergence of immune checkpoint inhibitors (ICIs) and various immunotherapeutic approaches (4–7). The use of ICIs has become vital in treating various cancers, offering a distinct survival advantage compared to traditional treatments, including chemotherapy and radiation (4–7). While conventional chemotherapy primarily targets cancerous cells to disrupt their cycle, ICIs consist of antibodies that target PD-1, PD-L1, or CTLA-4. This mechanism interrupts critical regulatory signals that inhibit immune activity in the tumor microenvironment (TME) (4–8). As a result, ICIs reduce immune suppression, allowing tumor-specific T cells to trigger an antitumor response by leveraging the patient’s immune capabilities against the cancer (4–7).

The efficacy of ICI treatment varies significantly among different cancer types, typically ranging from 10% to 40%. Most patients ultimately experience progression despite an initial favorable response (9, 10). Furthermore, the negative effects associated with immune responses triggered by ICI treatment can be severe or even life-threatening (11). Consequently, early identification of patients unlikely to benefit from ICI therapy has become a critical focus in oncology, aimed at preventing ineffective therapy and reducing the risk of adverse reactions (12, 13). At present, intra-tumoral PD-L1 assays are commonly employed as biomarkers to inform ICI treatment (14, 15). However, the clinical predictive value of PD-L1 in practice is limited due to its heterogeneous expression across tumor tissues (16). Additional immune-related biomarkers, such as tumor mutation burden, are also used for companion diagnostics (17–20). Nevertheless, the individual effectiveness of these markers in predicting treatment outcomes is restricted (17, 18). Moreover, establishing standardized criteria for the quantification of these biomarkers presents significant challenges. Thus, the discovery of new prognostic markers that can improve outcomes for cancer patients receiving ICIs is critically important.

The spleen is the largest lymphoid organ in humans, containing diverse populations of immune cells. Previous studies suggest that individuals with splenomegaly may experience splenic dysfunction along with alterations in the immune microenvironment. This abnormal enlargement could potentially affect the efficacy of ICIs, influenced by imbalances in the immune microenvironment. However, the predictive effect of baseline spleen volume or changes in spleen volume on the efficacy of ICI in cancer patients remains controversial. This study aims to offer valuable insights by systematically consolidating all existing evidence, thereby improving our comprehension of the clinical significance of spleen volume in forecasting the prognosis of cancer patients treated with ICIs. To the best of our knowledge, this represents the first meta-analysis examining the role of spleen volume in predicting prognosis in cancer patients receiving ICI.

## 2 Methods

### 2.1 Study cohort and data collection

The institutional review board approved this study. Due to its retrospective nature, informed consent was not required. We conducted a retrospective analysis of patients with hepatocellular carcinoma (HCC) who received immunotherapy and angiogenesis blockade therapy at our institution between December 2020 and June 2022. The immunotherapy treatments included anti-PD-1 and anti-PD-L1 agents. Eligible patients had diagnosed HCC, with a baseline computed tomography (CT) scan performed within four weeks before the start of treatment. Patients were included if they had at least one measurable lesion, as defined by RECIST version 1.1. Exclusion criteria included prior immunotherapy exposure and the absence of a pretreatment CT scan.

Comprehensive data were collected from patient medical records, encompassing demographic details (age, sex), Eastern Cooperative Oncology Group performance status (ECOG PS), hepatitis etiology, liver cirrhosis, Barcelona Clinic Liver Cancer (BCLC) classification, Child–Pugh classification, tumor count, macrovascular invasion, treatment line, modified albumin-bilirubin grade, and AFP levels. Tumor progression was assessed according to RECIST version 1.1. Follow-up CT imaging was performed every one to three months after the initiation of treatment. Progression-free survival (PFS) was defined as the duration from the start of ICI therapy to death or disease progression, while overall survival (OS) was measured from the start of ICI therapy until death.

### 2.2 Spleen volume estimation

Spleen volume was assessed using CT imaging, following the method outlined in a previous study (21). The spleen’s maximal width (W) was determined by measuring the largest diameter across any transverse section, while the thickness at the hilum (Th) was defined as the distance between the inner and outer borders of the spleen on a plane perpendicular to the width and intersecting the hilum. Additionally, the spleen length (L) was recorded from abdominal CT scans. Spleen volume was calculated using the following formula: Spleen volume = 30 + 0.58 (W × L × Th).

### 2.3 Search strategy and inclusion/exclusion criteria

An electronic search was initiated on February 1, 2025, across several bibliographic databases, including PubMed, EMBASE, and the Cochrane Library. The search incorporated a range of key terms such as “immune checkpoint inhibitors” [Mesh], “PD-1 inhibitors,” “PD-L1 inhibitors,” “CTLA-4 inhibitors,” “splenomegaly” [Mesh], “splenic volume,” “spleen volume,” and “enlarged spleen,” with a focus on all relevant fields. Only studies published in English involving human participants were included. Detailed search strategies are outlined in **Supplementary Material 1**. In addition, grey literature was sourced from Google Scholar, and reference lists of eligible studies were manually reviewed. All search results, both electronic and manual, were consolidated in Covidence software for streamlined data management, in accordance with Cochrane collaboration guidelines.

Inclusion criteria were as follows: (i) studies involving cancer patients, (ii) administration of ICIs as the therapeutic approach, (iii) evaluation of the association between baseline spleen volume (categorized into high and low groups) and changes in spleen volume and prognosis, and (iv) documentation of at least one outcome measure, including OS or PFS. Exclusion criteria included studies utilizing methodologies such as animal models, literature reviews, case reports, or conference abstracts and studies lacking hazard ratios (HRs) for evaluating outcomes based on published or text data. In cases of overlapping patient cohorts, preference was given to studies that provided comprehensive data and adhered to rigorous research methodologies.

### 2.4 Data extraction and quality assessment

During the data collection phase, we extracted key details such as author information, year of publication, study period, country, cancer type, treatment regimens, sample size, and gender, along with splenic volume-related parameters (including measurement tool and calculation method). HRs and their respective 95% confidence intervals (CIs) were primarily obtained from multivariate analyses. In cases where these were unavailable, data were derived from univariate analyses or extracted from survival curves using Engauge Digitizer software (22).

To evaluate the quality of the included observational studies, the Newcastle-Ottawa Scale (NOS) was used, with studies scoring six or more points classified as high quality (23). The quality assessment considered nine criteria across three domains: patient selection, study comparability, and outcome evaluation. All aspects of the process, including literature retrieval, screening, data extraction, and quality assessment, were independently performed by two researchers, with any disagreements resolved through discussions with the senior author.

Two independent reviewers conducted the screening and data extraction processes to reduce bias and ensure accuracy. Any discrepancies were resolved through discussion or consultation with a third reviewer.

### 2.5 Statistical methods

The Cox proportional-hazards model and the Kaplan-Meier method were used to assess survival curves across different groups. Meta-analysis was conducted using Stata version 18.0, with results visually represented through forest plots. Heterogeneity was assessed using Cochran’s Q test and the I² statistic, with significant variation defined as a *p*-value < 0.1 or an I² value > 50%. In cases of significant heterogeneity, a random-effects model based on the DerSimonian-Laird approach was applied; otherwise, a fixed-effects model using the Inverse Variance method was used. Publication bias was evaluated through Egger’s regression test (24) and Begg’s test (25). Sensitivity analyses were performed to test the robustness of the findings by sequentially excluding individual studies (26). Additionally, subgroup analyses were conducted based on different body composition assessment techniques. Statistical significance was set at a two-tailed *p*-value of < 0.05.

## 3 Results

### 3.1 Patient characteristics

**Table 1** outlines the characteristics of the patient cohort. The median age was 61.3 years, with a range from 42.5 to 83.2 years. Within the cohort, 38 patients (62.3%) were male. The ECOG PS was 0 in 39 patients (63.93%) and 1 in 22 patients (36.07%). Viral infections were present in 48 individuals (78.69%), and liver cirrhosis was identified in 41 patients (67.21%). The distribution of Barcelona Clinic Liver Cancer stages was as follows: early stage (n=3, 4.92%), intermediate stage (n=26, 42.62%), and advanced stage (n=32, 52.46%).

**Table 1:** Patient characteristics.

Factors	Overall (n=61)
Age	61.3 (42.5-83.2)
Males	38 (62.30%)
ECOG PS	
0	39 (63.93%)
1	22 (36.07%)
Etiology	
Viral	48 (78.69%)
Other	13 (21.31%)
Liver cirrhosis	
Yes	41 (67.21%)
No	20 (32.79%)
BCLC stage	
Early	3 (4.92%)
Intermediate	26 (42.62%)
Advanced	32 (52.46%)
Child-Pugh class	
A	51 (83.61%)
B	10 (16.39%)
Tumor number	
< 3	46 (75.41%)
≥ 3	15 (24.59%)
Macrovascular invasion	
Yes	18 (29.51%)
No	43 (70.49%)
Treatment line	
First-line	35 (57.38%)
Later-line	26 (42.62%)
mALBI grade	
1	28 (45.90%)
2	33 (54.10%)
AFP (ng/mL)	
≥ 400	34 (55.74%)
< 400	27 (44.26%)

Data shown are means with range or numbers with percentage.

ECOG PS, Eastern Cooperative Oncology Group performance status; BCLC, Barcelona Clinic Liver Cancer; AFP, α-fetoprotein; mALBI grade, modified albumin-bilirubin grade.

### 3.2 Relationship between baseline splenic volume, changes in splenic volume, and prognosis

**Figure 1:**
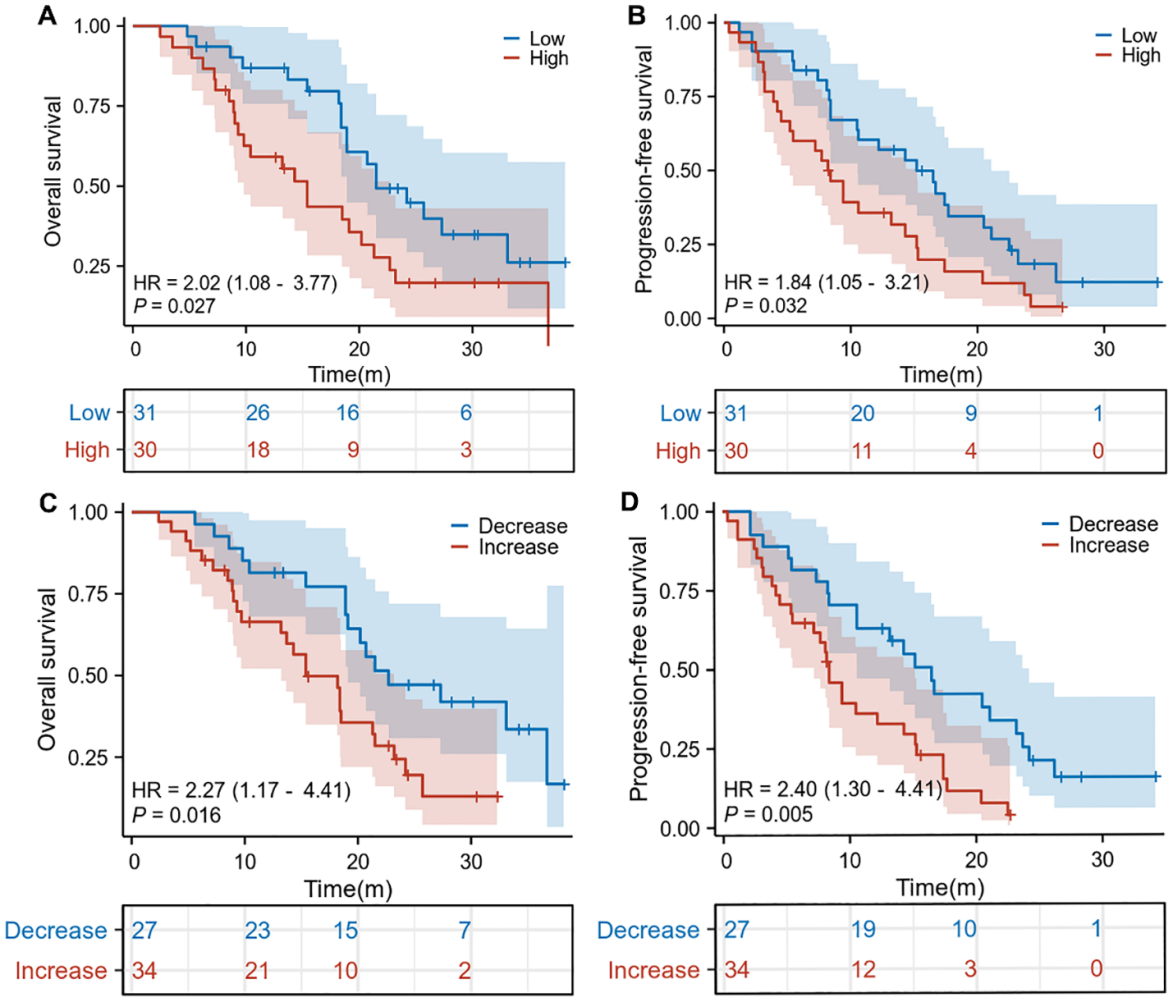
The Kaplan-Meier curve of overall survival **(A)** and progression-free survival **(B)** in the pretreatment high splenic volume and low splenic volume in our cohorts. The Kaplan-Meier curve of overall survival **(C)** and progression-free survival **(D)** according to the relative change compared to baseline splenic volume in our cohorts. HR, hazard ratio; CI, confidence interval.

The median basal splenic volume of all patients was 198 mL (range: 118–369). We divided the cohort into two groups based on the cutoff value for the median pretreatment splenic volume. Survival curves revealed significantly shorter OS (HR: 2.02, 95% CI: 1.08–3.77, *p* = 0.027, **Figure 1A**) and PFS (HR: 1.84, 95% CI: 1.05–3.21, *p* = 0.032; **Figure 1B**) in HCC patients with high baseline spleen volume compared to those with low spleen volume. Additionally, we also found that HCC patients with an increase in spleen volume during ICI treatment exhibited worse OS (HR: 2.27, 95% CI: 1.17–4.41, *p* = 0.016; **Figure 1C**) and PFS (HR: 2.40, 95% CI: 1.30–4.41, *p* = 0.005; **Figure 1D**) compared to those with a decrease in spleen volume. Therefore, data from our center indicate that HCC patients with high baseline spleen volume or an increase in spleen volume during ICI treatment have a worse prognosis.

### 3.3 Search results and included studies

**Figure 2:**
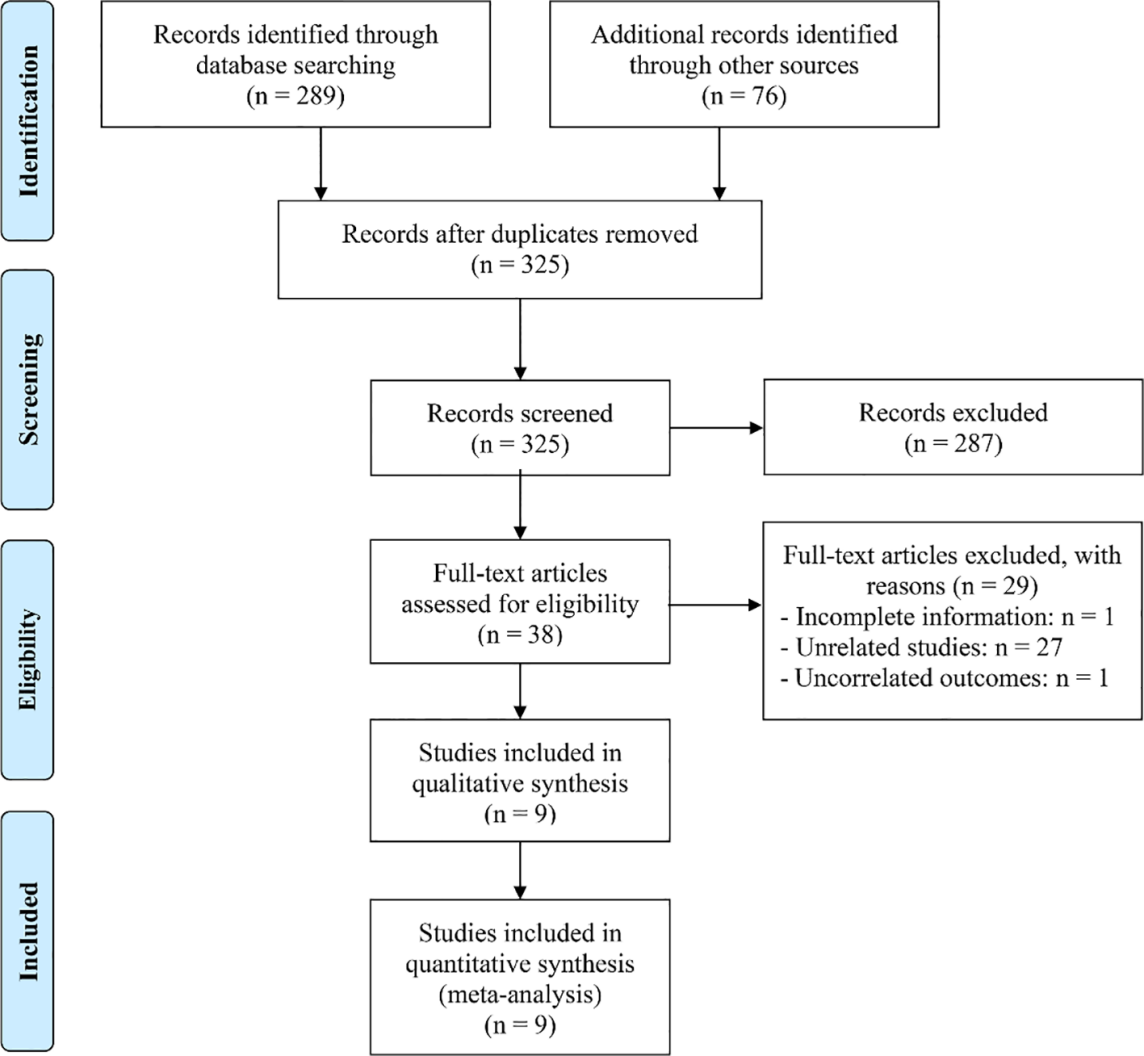
The flow diagram of identifying eligible studies.

The initial search strategy, combined with manual screening, identified 365 potentially relevant articles. After removing 40 duplicates, 287 articles were excluded based on title and abstract screening for non-compliance with the selection criteria. A full-text evaluation of the remaining 38 articles resulted in the exclusion of 29 that did not meet the required standards. Ultimately, 9 studies (27–35) were included in the final analysis (**Figure 2**).

### 3.4 Study characteristics

**Table 2** summarizes the key features of the studies analyzed in this research. A total of 1,024 participants were included, of whom 77.34% were male, with individual study sample sizes ranging from 45 to 276. Geographically, three studies were conducted in China, two in Germany, and one each in Brazil, France, Japan, and Turkey. To evaluate spleen volume, all studies employed either computed tomography (CT) or magnetic resonance imaging (MRI). These studies were exclusively retrospective in design, with NOS scores ranging from 6 to 8, indicating a low likelihood of bias (**Table 1**).

**Table 2:** Main characteristics of the studies included.

Study	Study period	Country	Sample size	Gender (male/female)	Treatment	Cancer type	Tool	Calculation method	NOS
Hatanaka et al., 2024 (34)	09/2020-07/2022	Japan	164	135/29	Atezo/bev	HCC	CT	30 + 0.58 (W × L × Th)	8
Mo et al., 2024 (35)	03/2016-12/2022	China	168	136/32	Atezo/bev or bevacizumab+sintilimab or camrelizumab	HCC	CT	30 + 0.58 (W × L × Th)	8
Oliveira et al., 2023 (33)	09/2019-03/2021	Brazil	50	32/18	Nivolumab or pembrolizumab or ipilimumab+nivolumab	Melanoma	CT or MRI	Fully automated AI-based splenic segmentation	7
Duwe et al., 2023 (32)	01/2012-05/2022	Germany	65	50/15	ICIs	UC, RCC	CT or MRI	Fully automated AI-based splenic segmentation	6
Xiao et al., 2022 (30)	08/2018-10/2020	China	161	144/17	ICIs	PLC	CT	Maximum diameter of the spleen	7
Yang et al., 2021 (28)	06/2015-05/2020	China	45	31/14	Nivolumab	PA	CT or MRI	30 + 0.58 (W × L × Th)	6
Müller et al., 2022 (29)	05/2016-10/2021	Germany	50	40/10	Atezo/bev or pembrolizumab or nivolumab	HCC	CT	Fully automated AI-based splenic segmentation	6
Galland et al., 2021 (27)	01/2014-06/2020	France	276	193/83	Anti-PD-1/PD-L1	NSCLC	CT	30 + 0.58 (W × L × Th)	8
Aslan et al., 2023 (31)	09/2010-09/2021	Turkey	45	31/14	Nivolumab	RCC	CT	30 + 0.58 (W × L × Th)	6

Atezo/bev: atezolizumab+bevazizumab; ICIs, immune checkpoint inhibitors; PD-1, programmed cell death protein 1; PD-L1, programmed cell death ligand 1; HCC, hepatocellular carcinoma; UC, urothelial carcinoma; RCC, renal cell carcinoma; PLC, primary liver cancer; PA, pancreatic adenocarcinoma; NSCLC, non small cell lung cancer; CT, computed tomography; MRI, magnetic resonance imaging; W × L × Th, maximal width of the spleen × maximal thickness of the spleen × length of the spleen.

### 3.5 Baseline spleen volume and overall survival and progression-free survival

**Figure 3:**
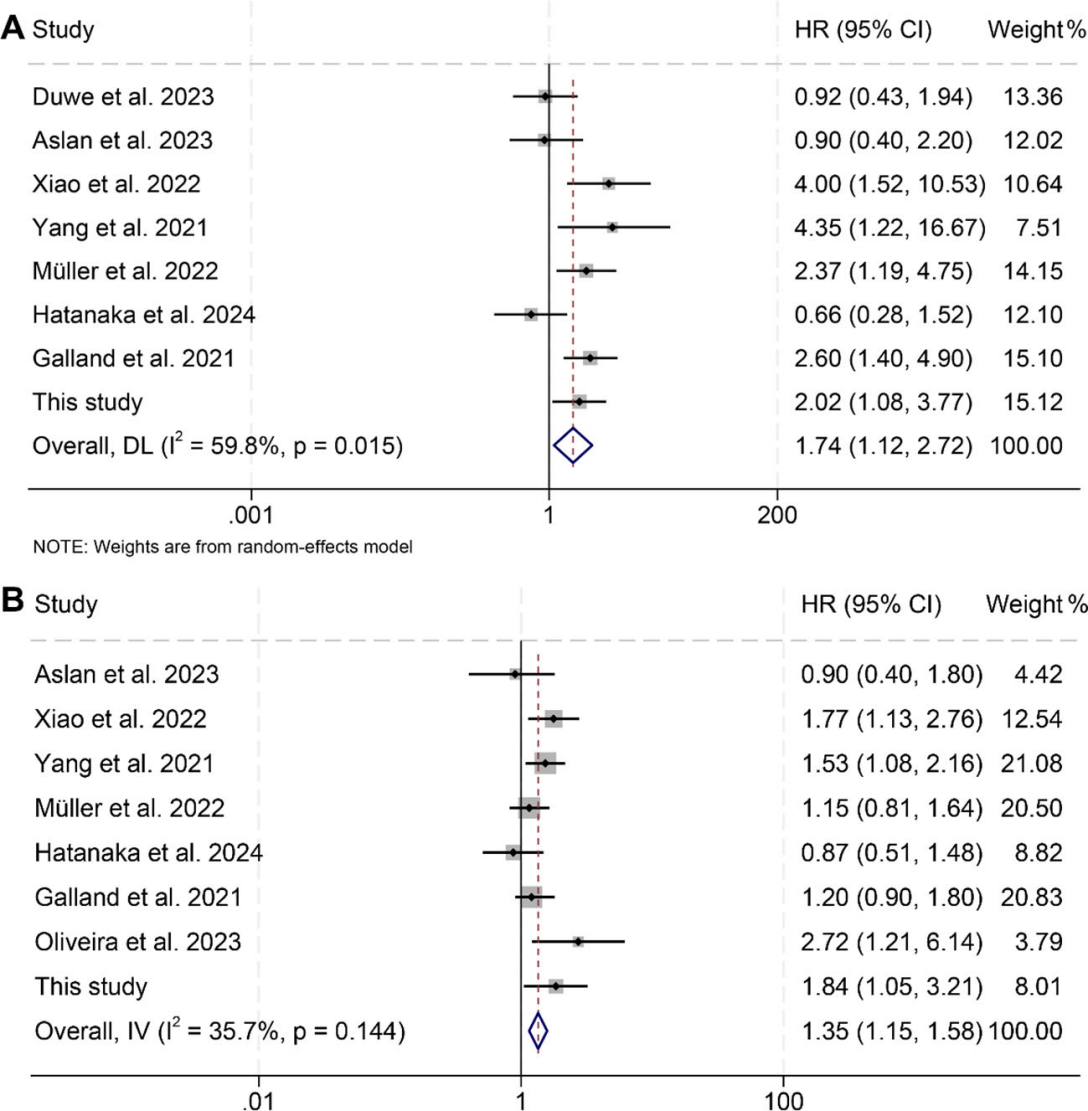
Forest plots illustrating the relationship between spleen volume and overall survival **(A)** and progression-free survival **(B)**. HR, hazard ratio; CI, confidence interval; DL, DerSimonian and Laird; IV, inverse-variance model.

In this investigation, we analyzed data from nine studies involving a total of 917 patients to assess the impact of pre-treatment spleen volumes on OS and PFS in cancer patients receiving ICIs. The results indicated that individuals with high spleen volume experienced significantly reduced OS (HR: 1.74, 95% CI: 1.12–2.72, *p* = 0.014, **Figure 3A**) and PFS (HR: 1.35, 95% CI: 1.15–1.58, *p* < 0.001, **Figure 3B**) compared to those with low spleen volume. The analysis of OS using the Cochran Q test and I² statistics (I² = 59.8%, p = 0.015) revealed considerable heterogeneity across the studies. Consequently, we employed a random-effects model for these analyses. In contrast, the evaluation of PFS studies did not show significant heterogeneity (I² = 35.7%, *p* = 0.144); thus, a fixed-effects model was considered appropriate.

**Figure 4:**
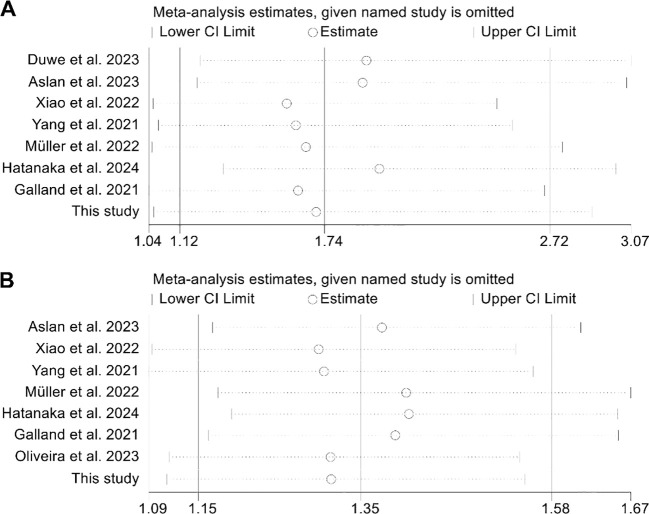
Sensitivity analysis of the relationship between spleen volume and overall survival **(A)** and progression-free survival **(B)**. HR, hazard ratio; CI, confidence interval.

The assessment of potential publication bias was performed using funnel plots, along with Begg’s and Egger’s tests. The results showed no significant bias concerning OS (Egger’s test: *p* = 0.913; Begg’s test: *p* = 0.711; **Supplementary Figure S1A**) or PFS (Egger’s test: *p* = 0.651; Begg’s test: *p* = 0.902; **Supplementary Figure S1B**). Our sensitivity analysis, which systematically excluded each study one at a time, demonstrated the consistent stability and robustness of the pooled HRs for both OS and PFS (**Figures 4A, B**).

### 3.6 Changes in splenic volume and overall survival and progression-free survival

**Figure 5:**
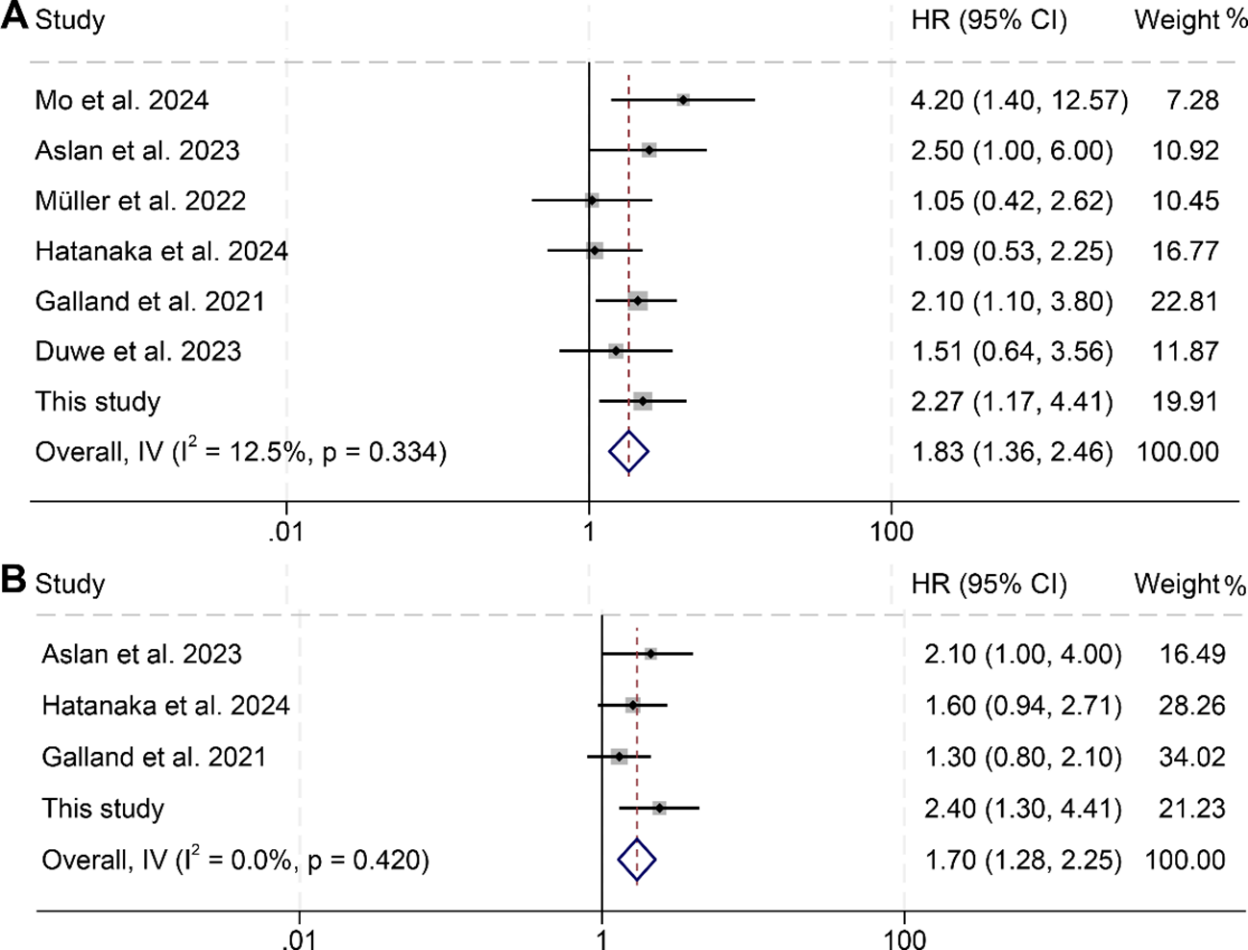
Forest plots illustrating the relationship between changes in splenic volume and overall survival **(A)** as well as progression-free survival **(B)**. HR, hazard ratio; CI, confidence interval; IV, inverse-variance model.

An analysis was further conducted to investigate the correlation between changes in splenic volume and OS as well as PFS among cancer patients, incorporating data from seven studies with a total of 829 subjects. Notably, these studies exhibited minimal heterogeneity for both OS (I² = 12.5%, *p* = 0.334) and PFS (I² = 0, *p* = 0.420), supporting the use of a fixed-effects model for the analysis. The results clearly indicated that patients with increases in splenic volume faced significantly worse outcomes, with reduced OS (HR: 1.83, 95% CI: 1.36–2.46, *p* < 0.001, **Figure 5A**) and PFS (HR: 1.70, 95% CI: 1.28–2.25, *p* < 0.001, **Figure 5B**) compared to those with decreases in splenic volume.

**Figure 6:**
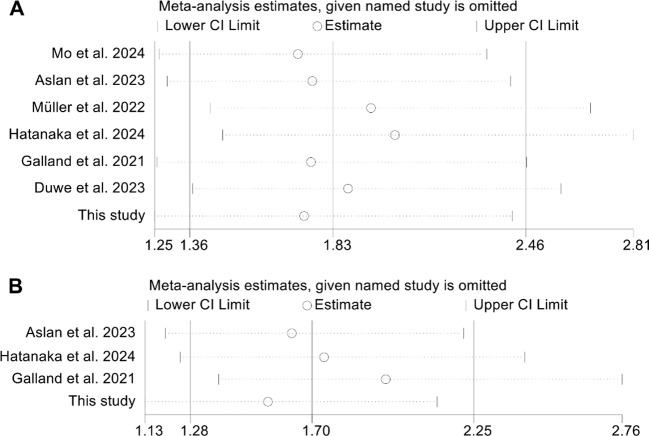
Sensitivity analysis of the relationship between changes in splenic volume and overall survival **(A)** and progression-free survival **(B)**. HR, hazard ratio; CI, confidence interval.

Publication bias was assessed using funnel plots, along with Begg’s and Egger’s tests. The findings showed no significant bias for OS (Egger’s test: *p* = 0.753; Begg’s test: *p* = 0.548; **Supplementary Figure S2A**) or PFS (Egger’s test: *p* = 0.129; Begg’s test: *p* = 0.308; **Supplementary Figure S2B**). Additionally, sensitivity analysis, which sequentially excluded individual studies, reaffirmed the stability and robustness of the pooled HRs (**Figures 6A, B**).

## 4 Discussion

In our queue, we found that a higher baseline spleen volume and an increase in spleen volume during ICI therapy were predictors of a poor prognosis in HCC cancer patients treated with ICI. Considering the controversies between different studies, we further included nine studies for meta-analysis and the conclusions were consistent with the findings of our cohort.

The spleen plays a vital role in regulating hematopoiesis and immune responses, making it a key focus for assessing the effectiveness of immunotherapy across various cancer types (36). In animal models, a significant accumulation of myeloid-derived suppressor cells (MDSCs) has been noted in the spleen, resulting in splenomegaly (37, 38). Additionally, certain clinical studies have shown a correlation between MDSC levels and splenic volume (39). Measuring splenic volume is both quick and straightforward in clinical settings.

Recent studies have identified an increase in MDSCs as a significant factor contributing to resistance against immunotherapy (40). This heterogeneous group consists of immature, immunosuppressive myeloid progenitor cells. The prevalence of MDSCs is heightened in the spleen, bloodstream of cancer patients, and within the TME across various malignancies. Their elevation in these areas is influenced by chemokines, growth factors, and cytokines secreted by tumors (41, 42). MDSCs are known for their role in fostering immunotherapy resistance by suppressing the functions of natural killer cells and T cells, as well as by activating immunosuppressive regulatory T cells. These pathological cells create an immunosuppressive environment through the excessive production of interleukin-10, transforming growth factor-β, arginase, and nitric oxide within the TME. Additionally, they promote tumor progression by expressing surface receptors that inhibit T cell activity (41, 43–46). Research shows that targeting and inactivating Tregs or MDSCs can restore the anticancer efficacy of ICIs (47–49). Furthermore, a study involving RCC indicated that combining MDSC-targeted therapy with IL-2 treatment enhances the response to immunotherapy (50). So far, the mechanism by which splenomegaly affects the curative effect of ICI has not been reported. We suggest that the relationship between splenomegaly and MDSCs may partly explain our conclusions.

Splenomegaly serves as a valuable predictor due to the straightforward, accessible, non-invasive, and cost-effective imaging methods available for assessing spleen size. Previous research indicated that high-affinity neoantigens are associated with improved OS in individuals diagnosed with HCC (51). However, the analysis of neoantigens was performed through whole-exome sequencing, a method that is both expensive and not readily available in clinical settings. Additionally, the expression of PD-L1 has been linked to the effectiveness of ICIs (52). Nonetheless, PD-L1 levels were assessed using immunohistochemistry, a technique that is invasive and not easily accessible in clinical practice. Another investigation found that immune-related adverse events could predict the effectiveness of ICIs (53). While these adverse events do not occur in every patient, spleen size can be reliably measured through imaging techniques for all individuals. Therefore, we believe that the assessment of spleen volume is a very meaningful indicator to predict the efficacy of ICI treatment.

The single-center cohort predominantly consisted of HCC patients with underlying cirrhosis, mainly of viral etiology, which is commonly associated with portal hypertension and resultant splenomegaly. Baseline splenomegaly in these patients may partly reflect the presence and severity of portal hypertension, a factor that could have independently contributed to poorer survival outcomes. We intentionally chose not to exclude these patients or set a mean baseline splenic volume cutoff in order to preserve the real-world clinical scenario, despite the potential confounding effect. Importantly, our meta-analysis, which incorporated a larger and more diverse patient cohort, supports the overall conclusions drawn from our findings. Nonetheless, future studies with more detailed stratification are warranted to further delineate the specific impact of portal hypertension and related factors on patient outcomes.

This meta-analysis has certain limitations that must be acknowledged. First, it is crucial to recognize that all studies included were retrospective cohort designs, which may restrict their statistical validity. Second, the limited number of studies analyzed hindered our ability to perform subgroup analyses for specific cancer types and ICIs. Third, the cut-off values for the same diagnostic criteria varied among the studies. Finally, due to the limited sample size in our single-center data, multivariate analysis was not performed. Therefore, to draw more robust conclusions, there is an urgent need for a global, multicenter study to explore the impact of splenic volume on the outcomes of cancer patients receiving ICIs.

## 5 Conclusion

A higher baseline spleen volume and an increase in spleen volume during ICI therapy were predictors of a poor prognosis in cancer patients treated with ICI.

## Data Availability

The original contributions presented in the study are included in the article/**Supplementary Material**. Further inquiries can be directed to the corresponding author/s.
