# Supplementary material for: Splenic volume as a predictor of survival in cancer patients treated with immune checkpoint inhibitors
Source: Front Immunol. 2025 May 30;16:1598484. doi: 10.3389/fimmu.2025.1598484 (PMC12163055; doi:10.3389/fimmu.2025.1598484)
Supplement: Supplementary file 1 [file DataSheet1.docx]

Supplementary material 1. Detailed article search strategy

((Camrelizumab) OR (Sintilimab) OR (Tislelizumab) OR (Toripalimab) OR (Envafolimab) OR (Immune Checkpoint Inhibitors) OR (Checkpoint Inhibitors, Immune) OR (Immune Checkpoint Inhibitor) OR (Checkpoint Inhibitor, Immune) OR (Immune Checkpoint Blockers) OR (Checkpoint Blockers, Immune) OR (Immune Checkpoint Blockade) OR (Checkpoint Blockade, Immune) OR (Immune Checkpoint Inhibition) OR (Checkpoint Inhibition, Immune) OR (PD-L1 Inhibitors) OR (PD L1 Inhibitors) OR (PD-L1 Inhibitor) OR (PD L1 Inhibitor) OR (Programmed Death-Ligand 1 Inhibitors) OR (Programmed Death Ligand 1 Inhibitors) OR (PD-1-PD-L1 Blockade) OR (Blockade, PD-1-PD-L1) OR (PD 1 PD L1 Blockade) OR (CTLA-4 Inhibitors) OR (CTLA 4 Inhibitors) OR (CTLA-4 Inhibitor) OR (CTLA 4 Inhibitor) OR (Cytotoxic T-Lymphocyte-Associated Protein 4 Inhibitors) OR (Cytotoxic T Lymphocyte Associated Protein 4 Inhibitors) OR (Cytotoxic T-Lymphocyte-Associated Protein 4 Inhibitor) OR (Cytotoxic T Lymphocyte Associated Protein 4 Inhibitor) OR (PD-1 Inhibitors) OR (PD-1 Inhibitor) OR (PD 1 Inhibitors) OR (Inhibitor, PD-1) OR (PD 1 Inhibitor) OR (Programmed Cell Death Protein 1 Inhibitor) OR (Programmed Cell Death Protein 1 Inhibitors) OR (Pembrolizumab) OR (Nivolumab) OR (Atezolizumab) OR (Ipilimumab) OR (Avelumab) OR (Tremelimumab) OR (Durvalumab) OR (Cemiplimab) OR (Immune Checkpoint Inhibitors[MeSH Terms])) AND ((Splenomegaly) OR (Enlarged Spleen) OR (Splenic volume) OR (Spleen volume) OR ("Splenomegaly"[Mesh]))


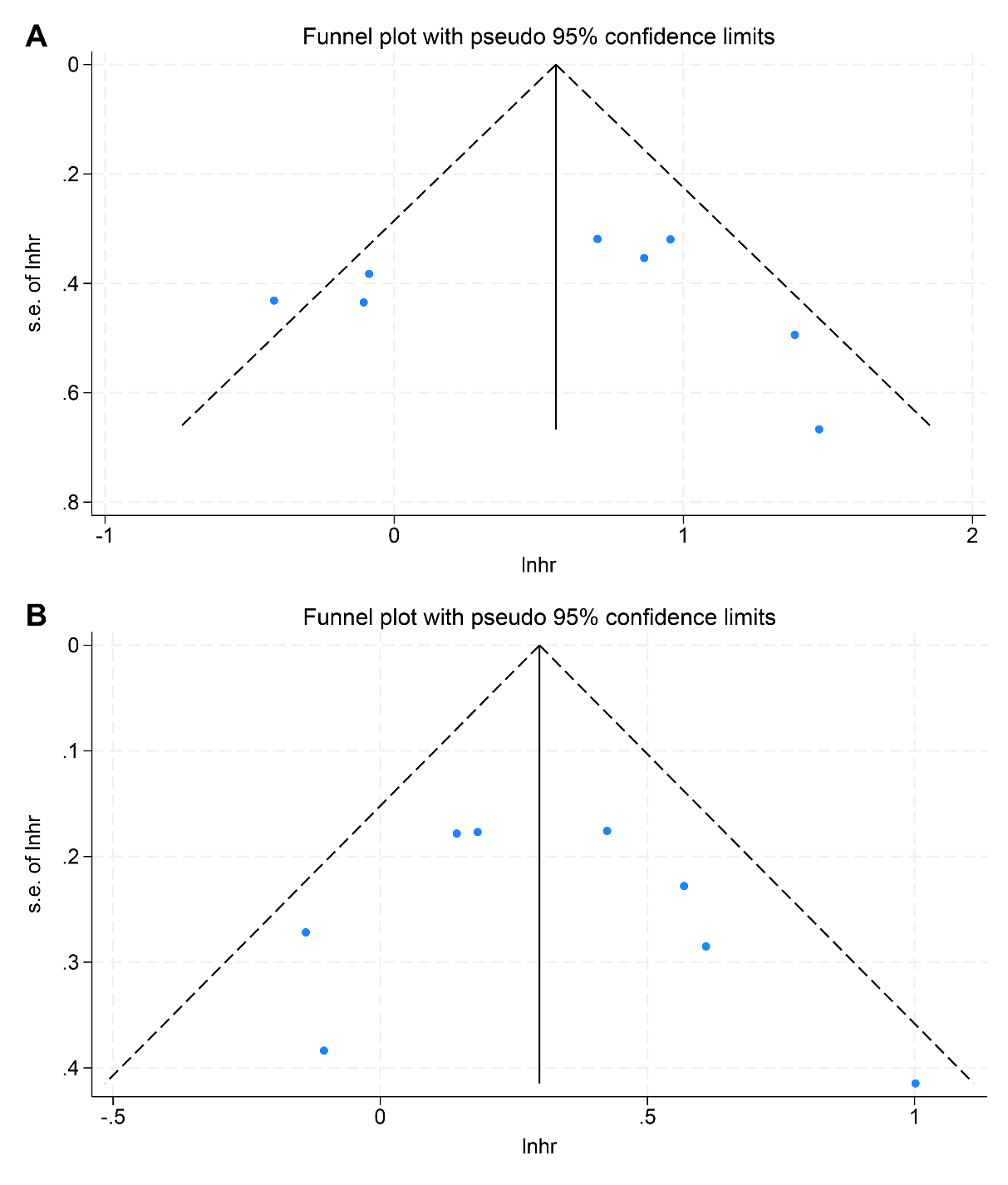


**Figure S1.** Funnel plots illustrating the relationship between the spleen volume and overall survival (A) as well as progression-free survival (B). HR, hazard ratio.


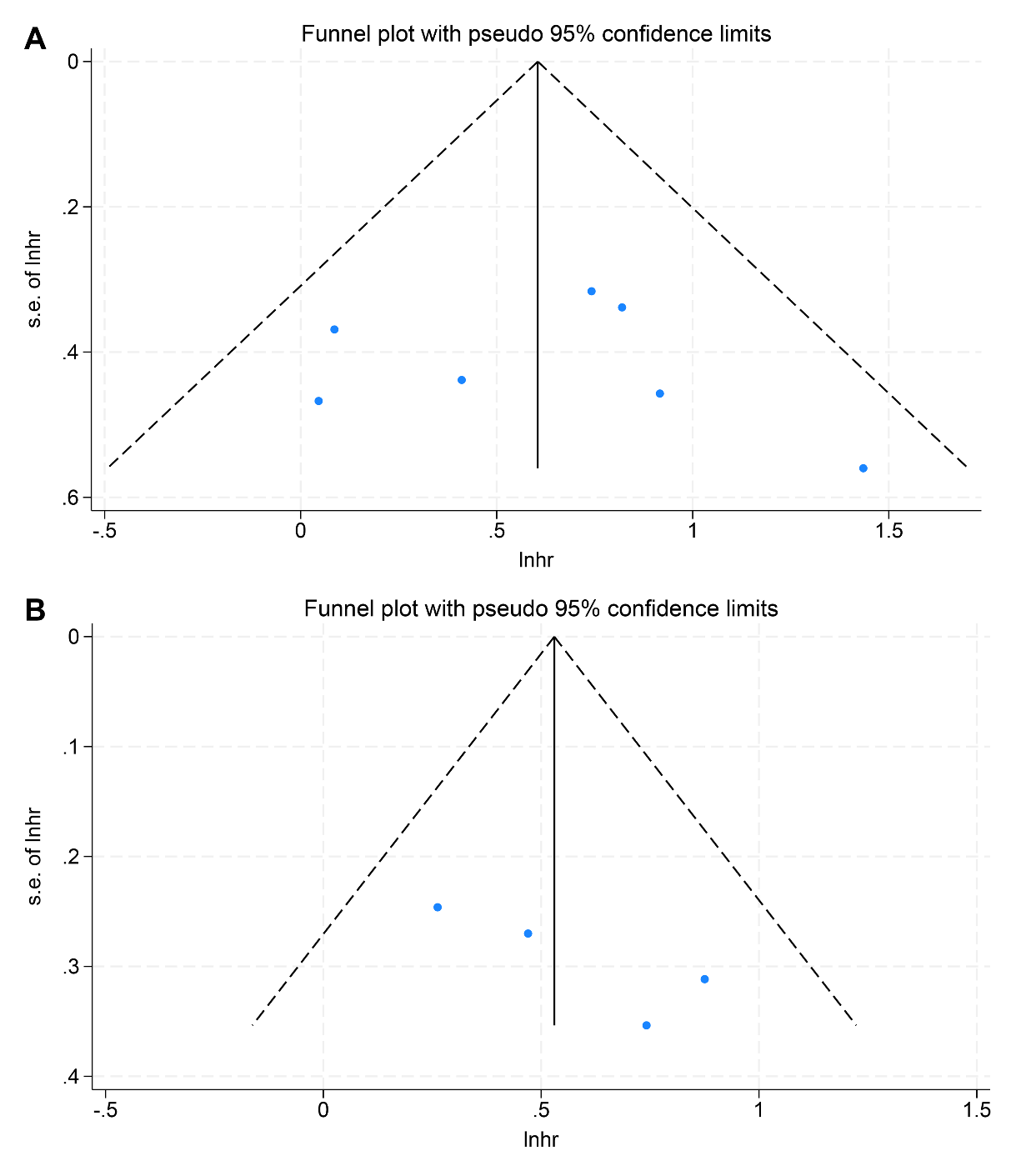


**Figure S2.** Funnel plots illustrating the relationship between changes in splenic volume and overall survival (A), as well as progression-free survival (B). HR, hazard ratio.
